# CD248 induces PD-L1 expression on cancer-associated fibroblasts to promote NSCLC immune escape

**DOI:** 10.3389/fcell.2025.1635915

**Published:** 2025-07-15

**Authors:** Zeyang Yang, Xuanyin Wang, Xu Zhu, Long Li, Xianling Zeng, Jiaming Ren, Lu Wang, Jiangwei Wu, Qiaoling Zhang, Siyu Wang, Maoqin Lu, Juan Zhai, Xinlei Liu, Jing Xiao, Tao Jin, Ying Zhang, Yun Wang, Jian Zhang, Zhu Zeng, Jieheng Wu

**Affiliations:** ^1^ Department of Immunology, Guizhou Medical University, Guiyang, China; ^2^ Department of Thoracic Surgery, The Affiliated Hospital of Guizhou Medical University, Guiyang, China; ^3^ School of Public Health, The Key Laboratory of Environmental Pollution Monitoring and Disease Control, Ministry of Education, Guizhou Medical University, Guiyang, China; ^4^ Guizhou Prenatal Diagnsis Center, The Affiliated Hospital of Guizhou Medical University, Guiyang, China; ^5^ Guizhou Key Laboratory of Microbio and Infectious Disease Prevention and Control, School of Basic Medical Sciences, Guizhou Medical University, Guiyang, China; ^6^ Immune Cells and Antibody Engineering Research Center of Guizhou Province, Key Laboratory of Biology and Medical Engineering, Guizhou Medical University, Guiyang, China; ^7^ The State Key Laboratory of Cancer Biology, Department of Biochemistry and Molecular Biology, The Fourth Military Medical University, Xi’an, China; ^8^ Tumor Immunotherapy Technology Engineering Research Center of Guizhou Medical University, Guizhou Medical University, Guiyang, China

**Keywords:** CD248, PD-L1, cancer-associated fibroblasts, non-small cell lung cancer, immune escape

## Abstract

**Background:**

Tumor immune escape is a critical step in tumor progression. Cancer-associated fibroblasts (CAFs) in the tumor microenvironment (TME) express abundant PD-L1 and suppress the functions of CD8^+^T cells, enablingd immune escape. CD248 is a candidate bioindicator for CAFs associated with non-small cell lung cancer (NSCLC), although its involvement in immune escape is not known.

**Methods:**

Fibroblasts were isolated from tumor and normal lung tissues from patients. We detected the expression of CD248 and PD-L1 on CAFs. Then, the influence of CAFs inhibited the function of CD8^+^T cells promoting NSCLC immune escape was assessed *in vivo* and *in vitro*. Finally, explored the mechanisms of which CD248 induced PD-L1 expression on CAFs.

**Results:**

Herein, we demonstrated that CD248 increased CAF PD-L1 levels, inhibiting CD8^+^T-cell function, thereby promoting NSCLC cell invasion and migration. CD248-induced FAK/Src/JNK/c-Jun axis activation promoted PD-L1 expression on CAFs. In tumor-bearing mice, lung tumors grew significantly slower, and the amount of granzyme B^+^CD8^+^T cells was greater in fibroblast-specific CD248 gene knockout mice than in wild-type mice. More importantly, we found that tislelizumab efficiency was improved in CD248 gene knockout mice.

**Conclusion:**

Our findings demonstrate that CD248 activates FAK/Src/JNK/c-Jun, thereby inducing PD-L1 expression on CAFs, which promotes NSCLC immune escape.

## 1 Introduction

Lung cancer (LC) is a common malignancy (Jachowski et al., 2023) that ranks second and first, respectively, in cancer morbidity and mortality rates. Non-small cell lung cancer (NSCLC) accounts for between 80% and 85% of LC cases (Siegel et al., 2024), representing a major global health challenge with high mortality (Hendriks et al., 2024). While small cell lung cancer (SCLC) represents approximately 15% of diagnoses and is characterized by aggressive growth, early metastasis, and strong neuroendocrine features (Rudin et al., 2021). Therapeutically, SCLC typically responds initially to platinum-based chemotherapy combined with immune checkpoint inhibitors (ICIs), but rapid relapse and acquired resistance remain major challenges (Horn et al., 2018; Huang et al., 2025). Tumor immune escape is a key event in cancer development (Ma et al., 2020). Immune checkpoint inhibitors (ICIs) have been introduced in the treatment of NSCLC to block the interaction between tumor cell-expressed PD-L1 and immune checkpoints, reactivating anti-tumor immune responses (Tang et al., 2022). Despite these advances, patients with LC continue to experience limited median and overall survival. Tumor immune escape remains a major driver of tumor recurrence and metastasis in LC (Liu and Cao, 2015; Giaj-Levra et al., 2020).

The PD-1, an immune checkpoint, and its associated ligand, PD-L1, synergistically facilitate tumor resistance to apoptosis and induce progression (Lei et al., 2020). The association between PD-1 and PD-L1 blocks T-cell activity (Jiang et al., 2019), suppressing T-cell activation (Liu et al., 2021). This mechanism hinders T cells from effectively eliminating tumor cells, allowing the cancer to evade immune surveillance. While much of the research has focused on PD-L1 expression by tumor cells, it is increasingly recognized that stromal cells can also contribute by expressing PD-L1 (Zou et al., 2022). Therefore, the issue of whether stromal cell PD-L1 can also induce tumor immune escape needs further research (Fang et al., 2021; Peng et al., 2019).

The tumor microenvironment (TME) contains tumor and stromal cells and extracellular matrix (Wang et al., 2022a). Cancer-associated fibroblasts (CAFs) contribute significantly to the TME (Mao et al., 2021). They promote tumor angiogenesis and immune escape and remodel the extracellular matrix, promoting tumor progression (Mao et al., 2021; Chen et al., 2021; Ma et al., 2022; Calvo et al., 2013). CAFs can induce immune escape by releasing numerous cytokines and chemokines (Monteran and Erez, 2019) and interacting with immune cells to affect cell differentiation and function. Furthermore, CAFs can impair CD8^+^ T-cell function, inhibiting their ability to eliminate tumor cells (Monteran and Erez, 2019). A high proportion of PD-L1-expressing CAFs indicates a worse prognosis in patients with esophageal cancer (Kawasaki et al., 2023). However, CAFs have a wide range of sources and are heterogeneous (Zhang et al., 2023), and the mechanism by which CAFs express PD-L1 to promote tumor immune escape remains unclear.

Tumor endothelial marker 1 (endosialin/TEM-1/CD248) is found in many cell types (Hong et al., 2022), including endothelial cells, cancer cells, and CAFs, and is associated with promoting tumorigenesis and tumor angiogenesis (Hong et al., 2022). Our previous study demonstrated that CD248-expressing CAFs contributed to cisplatin resistance in NSCLC by secreting IL-8 (Wu et al., 2024)^.^ Furthermore, CXCL12 produced by these fibroblasts facilitated the recruitment of M2 macrophages, promoting NSCLC progression (Wu et al., 2022a). These fibroblasts may also facilitate EMT in NSCLC by promoting the polarization of macrophages toward the M2 phenotype (Xiao et al., 2024). However, whether CAFs that express CD248 influence tumor immune escape remains unclear.

This study discovered that CD248, expressed in CAFs, activates the FAK/Src/JNK/c-Jun signaling pathway, leading to increased PD-L1 expression in CAFs. This, in turn, suppresses CD8^+^ T-cell activity and facilitates immune escape. These findings offer new mechanistic insight into how CAFs contribute to immune escape in NSCLC and highlight CD248 as a potential therapeutic target.

## 2 Materials and methods

### 2.1 Study approval

The collection of NSCLC and matched healthy tissue specimens was approved by the Clinical Research Ethics Committee of Guizhou Medical University and conducted following applicable ethical guidelines. Written informed consent was obtained from all participants. The study protocol (Approval No. 2021LL-52) received formal approval from the same ethics committee and adhered to all relevant ethical standards. All animal experiments were reviewed and approved by the Institutional Animal Care and Use Committee (IACUC) of Guizhou Medical University (Approval No. 2400397).

### 2.2 Human samples

NSCLC samples and corresponding normal tissues were collected from the Affiliated Hospital of Guizhou Medical University. The study was approved by the Ethics Committee of Guizhou Medical University (Approval No. 2021LL-52), and written informed consent was obtained from all participating patients.

### 2.3 Mice

C57BL/6 mice carrying either the *fsp-1*-Cre *transgene* or floxed *cd248* alleles were obtained from Suzhou Cyagen Co., Ltd. Conditional knockout (cKO) mice (*cd248* ^*fl/fl*^ *fsp-1* ^*cre*^ */* ^*+*^) were generated by crossing floxed *cd248* mice with *fsp-1*-Cre transgenic mice. In comparison, *cd248* ^*fl/fl*^ *fsp-1* ^*+/+*^ mice were used as wild-type (WT) controls. All animals were housed in a specific pathogen-free facility under standard conditions (12-h light/dark cycle, 22°C ± 1°C temperature, and 55% ± 5% relative humidity) with free access to food and water. Mice were individually housed and used for experiments between 6 and 12 weeks of age. Cre-negative littermates served as controls. All animal procedures were reviewed and approved by the Ethics Committee of Guizhou Medical University.

### 2.4 Cells and co-culture

The human NSCLC cell lines NCI-H460 and A549, along with the luciferase-expressing Lewis lung carcinoma (LLC-luciferase) cell line, were obtained from the American Type Culture Collection (ATCC) and confirmed to be free of *mycoplasma* contamination. Cells were cultured in RPMI-1640 or DMEM/F-12 medium (Gibco, United States) supplemented with 10% fetal bovine serum (FBS) and 1% penicillin-streptomycin (Invitrogen, United States) at 37°C in a humidified atmosphere containing 5% CO_2_. Fibroblasts were isolated and maintained using previously described methods (Wu et al., 2022b; Yang et al., 2020). Cells with stable CD248 silencing (CAFs-sh-CD248) or overexpression (CAFs-CD248OE) and control cells (CAFs-sh-CON) were grown via lentiviral transduction.

### 2.5 Assessment of migratory and invasive activity

To evaluate the migratory and invasive capabilities of cells, conditioned media (CM) derived from CAFs overexpressing CD248 (CAFs-CD248OE), CD248-silenced CAFs (CAFs-sh-CD248), or control CAFs (CAFs-sh-CON) were first used. For the Transwell assays, A549 and NCI-H460 cells were seeded into six-well plates and treated with CM from the respective CAF groups, followed by incubation in a humidified chamber for 48 h. In a separate experimental setup, CAFs-CD248OE, CAFs-sh-CD248, or CAFs-sh-CON were placed in the lower chambers of the Transwell system to assess migration and invasion directly. At the same time, A549 and NCI-H460 cells were seeded into the upper chambers and incubated for 48 h. All experiments were independently performed in triplicate.

### 2.6 Human CD8^+^ T-cell isolation assay

Human peripheral blood mononuclear cells (PBMCs) were obtained from the Affiliated Hospital of Guizhou Medical University for the isolation of CD8^+^T cells using a commercial isolation kit (Miltenyi Biotec, Germany; Cat. No. 130-096-495). The isolated CD8^+^ T cells were activated with a human CD3/CD28 T Cell Activator (STEMCELL Technologies, Canada; Cat. No. 10971). Activation efficiency was confirmed by flow cytometric analysis.

### 2.7 Western blotting

Proteins were extracted from cells using RIPA buffer, separated by SDS–polyacrylamide gel electrophoresis, and transferred onto PVDF membranes. The membranes were then probed with primary antibodies against CD248 (CST, #47948, United States), FAP (Servicebio, #GB11096), α-SMA (Servicebio, #GB11044), vimentin (Servicebio, #11192), FAK (Absin, #131894), Src (Servicebio, #GB11783), JNK (CST, #9252), c-Jun (CST, #9165), PD-L1 (CST, #86744), phospho-FAK (CST, #8556), phospho-JNK (CST, #4668), phospho-c-Jun (CST, #91952), and GAPDH (Servicebio, #11002). A horseradish peroxidase-conjugated secondary antibody (Servicebio) was used for detection, and protein bands were visualized using enhanced chemiluminescence (ECL) reagents (Thermo Fisher). Blots were sectioned before incubating with specific antibodies to optimize antibody use and conserve membrane material. Original images of blots images are presented in Supplementary Material, and a complete list of antibodies and their sources is provided in Supplementary Table S1.

### 2.8 Quantitative polymerase chain reaction (qPCR)

Total RNA was extracted using the MiniBEST Universal RNA Isolation Kit (Takara, Japan; #9767). Complementary DNA (cDNA) was synthesized using the PrimeScript RT Master Mix (Takara; #RR063A), and qPCR was performed with the TB Green Premix Ex Taq II Kit (Takara; #RR820). Primer sequences are listed in Supplementary Table S2.

### 2.9 Immunofluorescence (IF) staining

The IF analysis was conducted using the following primary antibodies: anti-human CD248 (CST, #47948), anti-α-SMA (Abcam, #ab5694), anti-PD-L1 (CST, #86744), and anti-c-Jun (CST, #9165). Multiplex immunofluorescence (mIF) staining combined with tyramide signal amplification (TSA) was used to evaluate the expression levels and colocalization of CD248 and PD-L1 in tissue sections. Further methodological details can be found in the research group’s previous published work (Wu et al., 2024).

### 2.10 Mouse tumor experiments

Animal experiments were designed and reported following the ARRIVE (Animal Research: Reporting of *In vivo* Experiments) guidelines. All procedures received approval from the Institutional Animal Care and Use Committee of Guizhou Medical University (Protocol No. 2400397) and complied with international standards for laboratory animal welfare. Fibroblast-specific CD248 knockout (cKO) mice established subcutaneous tumor-bearing mouse models. To model lung tumor progression, 5 × 10^5^ LLC-luciferase cells suspended in 200 µL of medium were injected subcutaneously into WT and cKO mice. Tumor growth was monitored using an *in vivo* fluorescence imaging system following intraperitoneal administration of D-luciferin and anesthesia with isoflurane. Image acquisition and analysis were performed using Living Image software. At the end of the experiment, mice were euthanized under isoflurane anesthesia, tumors were excised and imaged, and paraffin-embedded tissue sections were prepared for further analysis.

For the anti-PD-1 treatment experiment, subcutaneous tumor-bearing mouse models were established using fibroblast-specific CD248 knockout (cKO) mice. To mimic lung tumor progression, 5 × 10^5^ LLC-luciferase cells suspended in 200 µL of medium were injected subcutaneously into WT and cKO mice; PBS was the control. Mice received tislelizumab (BAIZEZAN^®^; BeiGene, Ltd, China. #G202306057), a humanized monoclonal antibody targeting PD-1 (anti-PD-1 antibody), at a dose of 5 mg/kg twice weekly. Tumor progression was monitored using an *in vivo* fluorescence imaging system, and data were analyzed using Living Image software. After the experiment, mice were euthanized under isoflurane anesthesia, tumors were collected and imaged, and paraffin-embedded sections were prepared for further histological analysis.

### 2.11 Flow cytometry

For CD8^+^T cell detection assays, CD8^+^T cells were co-cultured with either CAFs-sh-CD248 or CAFs-sh-CON for 48 h, then stained with a FITC-conjugated anti-CD8 antibody (BioLegend, #344704) and a PE-conjugated anti-granzyme B antibody (BioLegend, #372208) for 30 min at 4°C in the dark. Flow cytometry was used for subsequent analysis.

For tumor-infiltrating CD8^+^T cell analysis, tumor tissues were excised, mechanically dissociated, and enzymatically digested using 50 U/mL collagenase I (Absin, #abs47048000) for 3 h at 37°C. The resulting cell suspensions were passed through a 40-μm filter and subjected to red blood cell lysis. After washing twice with cold PBS, cells were incubated for 30 min at 4°C in the dark with FITC-conjugated anti-mouse CD8b.2 (BD BioLegend, #140403) and PE-conjugated anti-granzyme B antibodies. Samples were then analyzed via flow cytometry.

### 2.12 Co-immunoprecipitation (Co-IP)

According to the manufacturer’s instructions, the Co-IP was performed using a commercial kit (Absin, #abs9649). Cell lysates were incubated with the designated primary antibodies for 1 h, followed by an overnight incubation at 4°C with antibody-conjugated beads. After thorough washing, the samples were processed for protein detection. Antibodies against FAK, JNK, and rabbit IgG (used at 2 µg per mg of total protein) were used to identify the target proteins.

### 2.13 Statistical analysis

All data analyses were conducted using Excel 2016. Before statistical evaluation, normality and homogeneity of variance were assessed. Results are presented as mean ± SD or mean ± SEM, and comparisons between groups were made using two-tailed t-tests. A p-value of less than 0.05 was considered statistically significant.

## 3 Results

### 3.1 CD248 levels in NSCLC-associated CAFs

**FIGURE 1:**
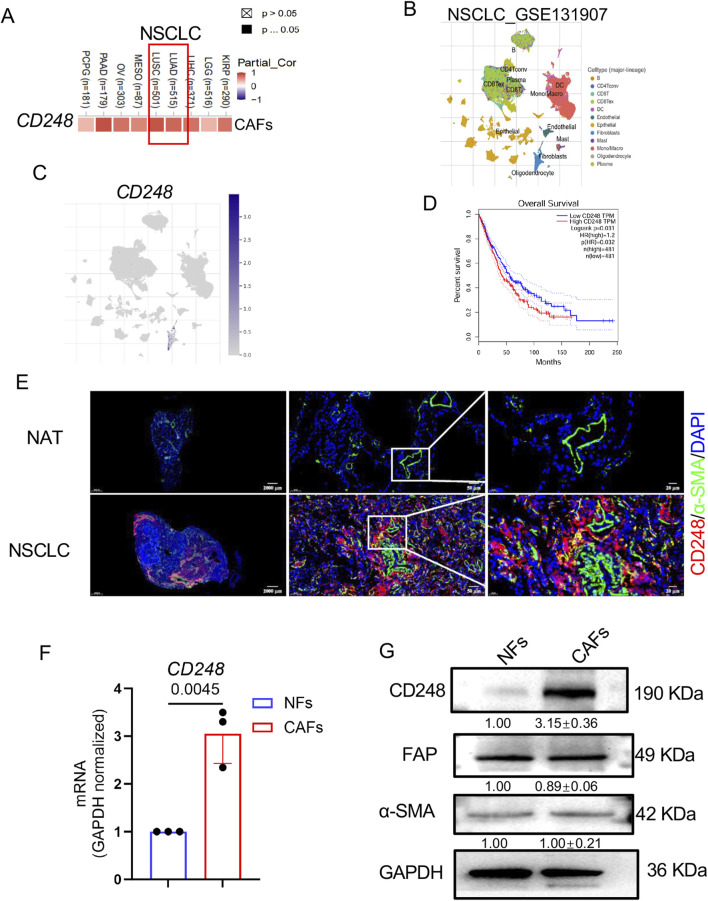
CD248 levels on NSCLC-derived CAFs. **(A)** CAFs CD248 levels in NSCLC, from TIMER 2.0. **(B,C)** Data from the TISCH database revealed distinct cell types in NSCLC and *CD248* expression in fibroblasts. **(D)** OS of NSCLC patients from the TCGA database. **(E)** Dual-IF staining showing α-SMA and CD248 in NSCLC and NAT tissues. Scale bars: right: 20 μm, middle: 50 μm, left: 2000 μm. **(F)** qPCR measurement of *CD248* levels in CAFs and NFs. Values are depicted as means ± SD from 3 experiments. **(G)** CD248, FAP, and α-SMA protein levels were shown by Western blotting and quantified with ImageJ. Values are mean ± SD from 3 experiments.

The CD248 expression was analyzed using the TIMER 2.0 database, revealing a strong positive correlation with the proportion of CAFs (Figure 1A). Further analysis using the Tumor Immune Single-Cell Hub (TISCH) database showed that CD248 expression was predominantly restricted to CAF populations in NSCLC samples (Figures 1B,C). Data from The Cancer Genome Atlas (TCGA) indicated that elevated CD248 expression was significantly associated with poorer prognosis in NSCLC patients (Figure 1D). To validate these database findings, IF staining was performed, which demonstrated substantially higher CD248 expression in tumor tissues compared to adjacent normal tissues, with significant colocalization with α-SMA (Figure 1E). CAFs and normal fibroblasts (NFs) were then isolated from tumor and adjacent tissues, respectively. Both cell types expressed α-SMA and FAP, as confirmed by qPCR and Western blotting, but CD248 expression was markedly upregulated in CAFs (*p* = *0.0045*) (Figures 1F,G). These findings support a strong association between CD248 expression and CAFs in NSCLC.

### 3.2 PD-L1 was highly expressed on CD248^+^CAFs

**FIGURE 2:**
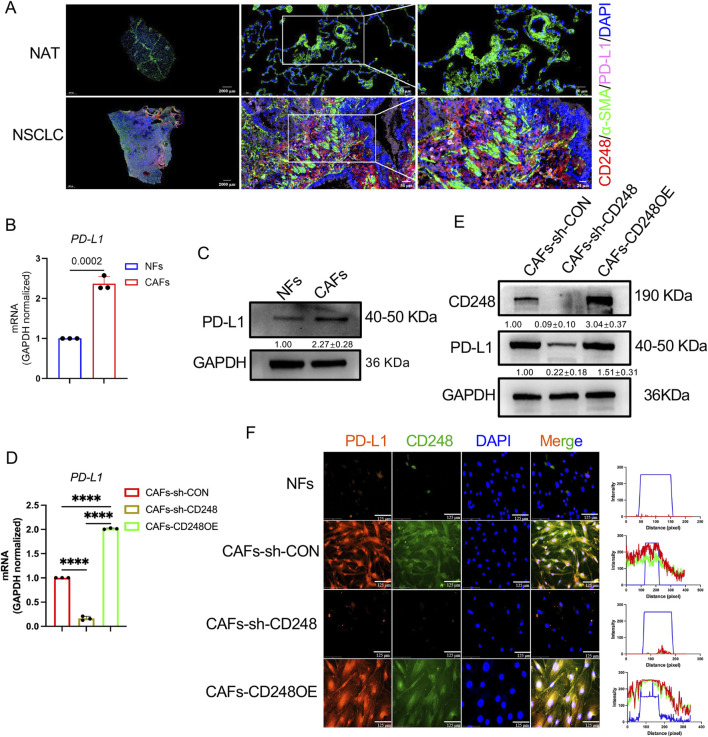
CD248^+^CAFs express high levels of PD-L1. **(A)** IF images showing CD248, α-SMA, and PD-L1 in tumor and normal tissues. Scale bars: right: 20 μm, middle: 50 μm, left: 2000 μm. **(B)** *PD-L1* expression in NFs and CAFs. Values are mean ± SD from 3 experiments. **(C)** PD-L1 protein levels in NFs and CAFs. Values are depicted as means ± SDs from 3 experiments. **(D)** PD-L1 in CAFs-CD248OE, CAFs-sh-CD248, and CAFs-sh-CON. Values are mean ± SD from 3 experiments. **(E)** CD248 and PD-L1 protein levels in CAFs-CD248OE, CAFs-sh-CD248, and CAFs-sh-CON. Values are depicted as means ± SDs from three separate experiments. **(F)** IF staining showing CD248 and PD-L1 in CAFs-CD248OE, NFs, CAFs-sh-CD248, and CAFs-sh-CON. Scale bar: 125 μm.

One study revealed that a high proportion of PD-L1-harboring CAFs indicates a worse prognosis among esophageal cancer patients (Kawasaki et al., 2023). To assess whether NSCLC-derived CAFs express PD-L1, IF staining was conducted to detect CD248 and PD-L1 in NSCLC tissues and NAT. PD-L1 expression was higher in NSCLC samples, with clear colocalization of CD248 and α-SMA (Figure 2A). qPCR analysis showed significantly elevated PD-L1 mRNA levels in CAFs compared to NFs (*p* = *0.0002*) (Figure 2B). These findings were further validated by Western blotting, which demonstrated increased PD-L1 protein expression in CAFs relative to NFs (Figure 2C).

It was observed that NSCLC-derived CAFs expressed CD248. To explore the relationship between CD248 and PD-L1 expression in CAFs, qPCR was performed on CAFs overexpressing CD248 (CAFs-CD248OE), CD248 knockdown CAFs (CAFs-sh-CD248), and control CAFs (CAFs-sh-CON). Compared to the control group, PD-L1 expression was significantly reduced in CAFs-sh-CD248 and elevated in CAFs-CD248OE (*p < 0.0001*) (Figure 2D). Western blot analysis further confirmed that CD248 knockdown decreased PD-L1 protein levels. At the same time, CD248 overexpression led to increased PD-L1 expression in CAFs (Figure 2E). Consistently, IF staining showed stronger PD-L1 signals in tissues containing CD248-overexpressing CAFs (Figure 2F). These results indicate that CD248^+^ CAFs upregulate PD-L1 expression.

### 3.3 CD248^+^CAFs enhance invasion and migration

**FIGURE 3:**
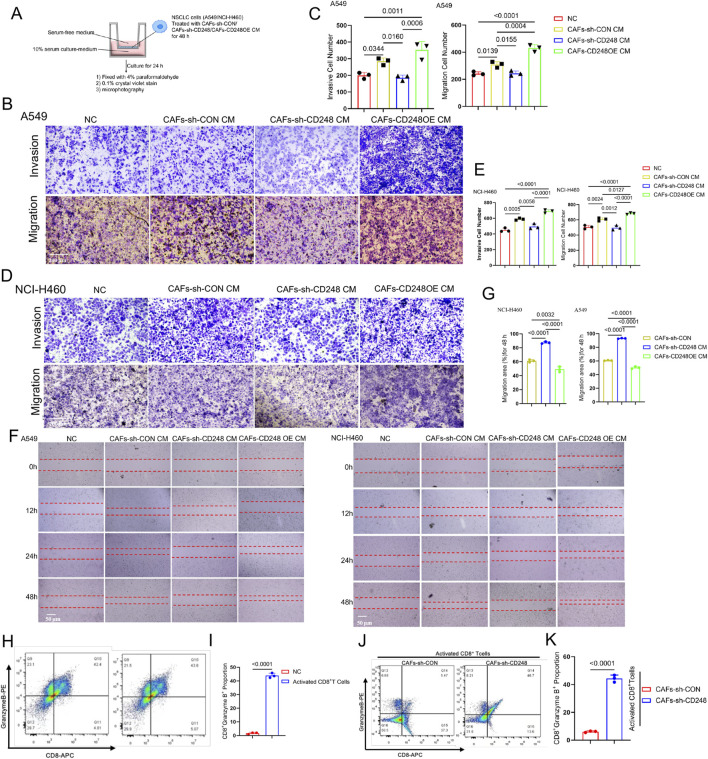
CD248^+^CAFs facilitate invasion and migration and inhibit the CD8^+^T lymphocyte activity. **(A–E)** Results of Transwell assays. NCI-H460 and A549 cells were grown together with CM from CAFs-CD248OE, CAFs-sh-CD248, or CAFs-sh-CON for 48 h, and then, the different CM-treated cells were resuspended in serum-free media before inoculation in the top compartment of the Transwell; alternately, the bottom compartment received complete media **(A)**. **(B)** Results of A549 cells after 24 h. Scale bar, 200 μm. **(C)** Invasive and migratory cell numbers. Values are mean ± SD from 3 experiments. **(D)** NCI-H460 cell analysis results after 24 h. Scale bar: 200 μm. **(E)** Migratory and invasive cell numbers. Values are mean ± SD from 3 experiments. **(F)** Results of scratch assays after treatment with CM from CAFs-CD248OE, CAFs-sh-CD248, or CAFs-sh-CON. Scale bar: 50 μm. **(G)** The migration area was assessed via ImageJ. Values are mean ± SD from 3 experiments. **(H)** Activated CD8^+^ T cell proportions from PBMCs. **(I)** Quantification of CD8^+^granzyme B^+^T cells. Values are mean ± SD from 3 experiments. **(J)** Proportion of activated CD8^+^T cells grown with CAFs-sh-CON or CAFs-sh-CD248 for 48 h. **(K)** The proportion of CD8^+^granzyme B^+^ T cells. Values are mean ± SD from 3 experiments.

To investigate whether CD248^+^CAFs contribute to tumorigenic behavior, NCI-H460, and A549 cells were cultured in CM derived from CAFs-CD248OE, CAFs-sh-CD248, or CAFs-sh-CON. Both migration and invasion capacities were significantly reduced in cells treated with CM from CAFs-sh-CD248. These properties were improved in cells exposed to CM from CAFs-CD248OE, compared to those treated with control CM (CAFs-sh-CON) (Figures 3A–E). Scratch assays further confirmed that cell migration was markedly impaired in the CAFs-sh-CD248 CM group (*P < 0.001*) (Figures 3F,G). These findings indicate that CD248^+^CAFs enhance the tumorigenic potential of NSCLC cells.

### 3.4 CD248^+^CAFs can inhibit CD8^+^T cell activity

It was confirmed that CD248^+^CAFs express PD-L1. To further assess whether PD-L1-expressing CD248^+^ CAFs suppress CD8^+^T cell activity, CD8^+^ T cells were isolated from PBMCs, successfully expanded, and activated using an anti-CD3/CD28 activator. Flow cytometry analysis revealed a significant increase in the activated CD8^+^ T cell population (CD8^+^granzyme B^+^) following stimulation with the CD3/CD28 activator (*P < 0.0001*) (Figures 3H,I). Subsequently, activated CD8^+^ T cells were co-cultured with either CAFs-sh-CON or CAFs-sh-CD248 for 48 h, followed by flow cytometric evaluation of CD8^+^granzyme B^+^ cells. Compared to the CAFs-sh-CON group, the population of activated CD8^+^ T cells was significantly increased in the CAFs-sh-CD248 group (*P < 0.0001*) (Figures 3J,K). These findings suggest that CD248^+^ CAFs attenuate CD8^+^ T cell cytotoxic activity.

### 3.5 CD248 activates FAK/Src/JNK/c-Jun to induce PD-L1 expression on CAFs

**FIGURE 4:**
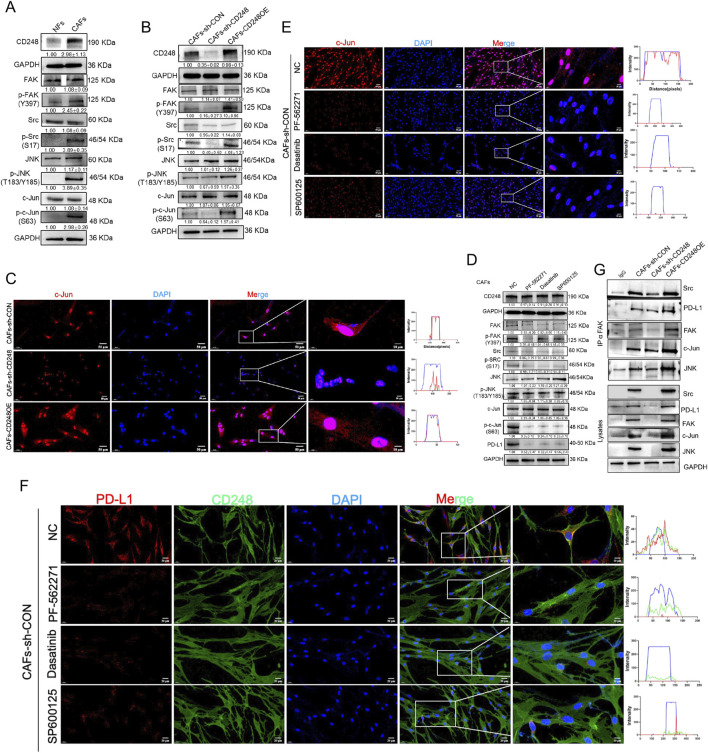
CD248 stimulates FAK/Src/JNK/c-Jun to enhance PD-L1 expression in CAFs. **(A)** Western blotting data show the CD248, FAK, p-FAK, Src, p-Src, JNK, p-JNK, c-Jun, and p-c-Jun expressions in NFs and CAFs, and relative expression was computed in Image J. Data are given as means±SDs from 3 experiments. **(B)** Western blotting data show the CD248, FAK, p-FAK, Src, p-Src, JNK, p-JNK, c-Jun, and p-c-Jun protein expressions in CAFs-CD248OE, CAFs-sh-CD248, and CAFs-sh-CON, and relative expression was computed in Image J. Values are mean ± SD from 3 experiments. **(C)** IF images showing the intranuclear localization of c-Jun in CAFs-CD248OE, CAFs-sh-CD248, and CAFs-sh-CON. Scale bars, 50 μm and 10 μm. **(D)** Western blotting data show the CD248, FAK, p-FAK, Src, p-Src, JNK, p-JNK, c-Jun, p-c-Jun and PD-L1 protein expressions in CAFs treated with inhibitors of FAK (PF-562271), Src (dasatinib) and JNK (SP600125), and relative expression was computed in Image J. Values are mean ± SD from 3 experiments. **(E)** IF staining depicting the intranuclear localization of c-Jun in CAFs-CD248OE, CAFs-sh-CD248, and CAFs-sh-CON treated with PF-562271, dasatinib and SP600125. Scale bars, 50 μm and 10 μm. **(F)** IF staining showing CD248 and PD-L1 in CAFs treated with PF-562271, dasatinib, and SP600125. Scale bars, 20 μm and 10 μm. **(G)** Associations between FAK and Src, JNK, c-Jun, and PD-L1 in the indicated CAFs were assessed using co-immunoprecipitation, and relative expressions were computed using Image J. Values are mean ± SD from 3 experiments.

A previous study has reported that FAK/Src can induce PD-L1 expression (Wei et al., 2021). FAK/Src can activate JNK/c-Jun (Mitra and Schlaepfer, 2006).And c-Jun can regulate the expression of PD-L1 (Cha et al., 2019; Goel et al., 2022).To investigate whether CD248 induces PD-L1 expression in CAFs through activation of the FAK/Src/JNK/c-Jun signaling pathway, the levels of FAK, Src, JNK, and c-Jun in CAFs-CD248OE, CAFs-sh-CD248, CAFs-sh-CON, and NFs were first analyzed using Western blotting. The results showed that phosphorylated FAK (p-FAK), Src (p-Src), JNK (p-JNK), and c-Jun (p-c-Jun) were significantly upregulated in CAFs-CD248OE compared to CAFs-sh-CON, NFs, and CAFs-sh-CD248 (Figures 4A,B). IF staining further revealed cytoplasmic localization of c-Jun in CAFs-sh-CD248 and predominant nuclear localization in CAFs-CD248OE (Figure 4C).

Next, CAFs were treated with specific inhibitors of FAK (PF-562271), Src (dasatinib), and JNK (SP600125). Western blotting demonstrated that treatment with any of these inhibitors significantly reduced the expression of p-FAK, p-Src, p-JNK, p-c-Jun, and PD-L1 (Figure 4D). Similarly, IF analysis showed decreased PD-L1 fluorescence intensity and cytoplasmic retention of c-Jun in inhibitor-treated CAFs compared to untreated controls (Figures 4E,F). Co-IP assays were conducted to determine whether FAK interacts directly with Src, JNK, c-Jun, and PD-L1 in CD248^+^ CAFs. The results confirmed that FAK is physically associated with Src, JNK, c-Jun, and PD-L1 in CD248-overexpressing CAFs (Figure 4G). These findings demonstrate that CD248 enhances PD-L1 expression in CAFs via the FAK/Src/JNK/c-Jun signaling cascade.

### 3.6 Fibroblast-specific *CD248* knockout inhibited NSCLC immune escape *in vivo*

**FIGURE 5:**
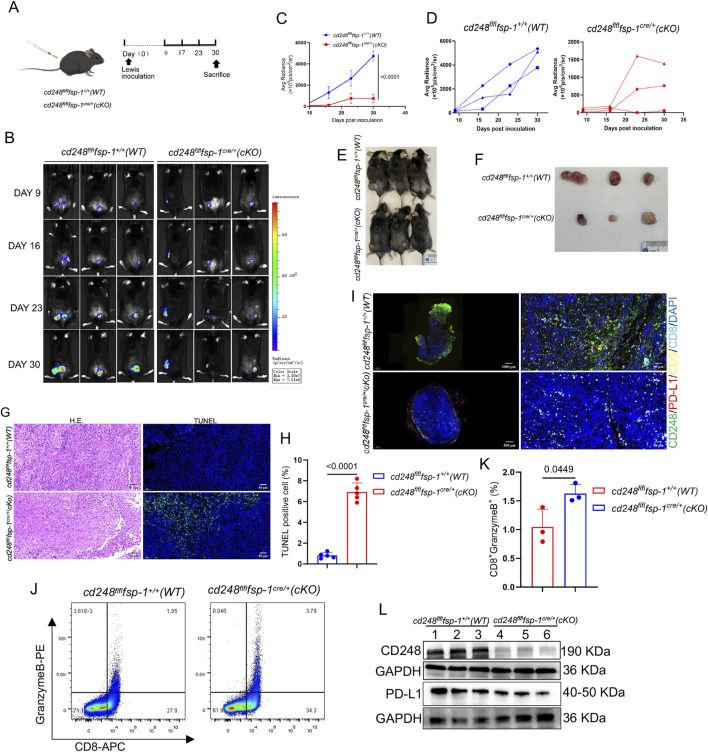
Fibroblast-specific *CD248* knockout inhibited tumor immune escape *in vivo*. Overall, 1 × 10^6^ LLC cells/200 μL were administered into *cd248* ^*fl/fl*^ *fsp-1* ^*+/+*^ (WT) and *cd248* ^*fl/fl*^ *fsp-1* ^*cre/+*^ (cKO) mice to generate a subcutaneous tumor-bearing model (n = 3). **(A)** A schematic diagram of the model. **(B)** Tumor growth, observed via BLI. **(C,D)** The average radiance intensity of each group was assessed. Values are depicted as mean ± SEM. **(E)** Mice were euthanized on completion of the experiment. **(F)** Images of tumor tissues from all groups of mice. **(G)** Tumor tissues were detected via H&E staining, and cell apoptosis was detected via IF staining for TUNEL (green). Scale bar, 50 μm. **(H)** The apoptotic tumor cell populations. Values are mean ± SEM. **(I)** IF images showing CD248 (green), PD-L1 (red), CD3 (yellow), and CD8 (cyan) colocalization in the tumor tissues of WT and cKO mice. Scale bars, 50 μm and 10 μm. **(J)** Flow cytometry analysis of CD8^+^granzyme B^+^T cell populations. **(K)** The CD8^+^granzyme B^+^T cell percentages were calculated (n = 3). Values are mean ± SEMs. **(L)** Evaluation of CD248 and PD-L1 protein expression in the tumors (n = 3 mice), as determined by Western blotting.

To evaluate whether CD248^+^ CAFs expressing PD-L1 contribute to immune evasion in NSCLC *in vivo*, LLC cells were subcutaneously implanted into *cd248* ^*fl/fl*^ *fsp-1* ^*+/+*^ (WT) and *cd248* ^*fl/fl*^ *fsp-1* ^*cre/+*^ (cKO) mice (Figure 5A). Tumor progression was tracked using bioluminescence imaging (BLI), and fluorescence intensity was quantified. Tumor volumes were significantly larger in WT mice compared to cKO mice (*p < 0.0001*) (Figures 5B–D), which was further validated upon dissection (Figures 5E,F).

Histological analysis of paraffin-embedded tumor sections using hematoxylin and eosin (H&E) and TUNEL staining showed fewer apoptotic cells in tumors from WT mice than those from cKO mice (*p < 0.0001*) (Figures 5G,H). IF staining for CD248, PD-L1, CD3, and CD8 revealed lower expression of CD248 and PD-L1 and greater infiltration of CD8^+^ T cells in tumors from cKO mice compared to WT controls (Figure 5I).

Single-cell suspensions of tumor tissues were further analyzed by flow cytometry, which demonstrated a significantly higher proportion of CD8^+^granzyme B^+^ T cells in cKO mice than in WT mice (*p = 0.0449*) (Figures 5J,K). Western blotting also confirmed reduced PD-L1 expression in tumors from cKO mice compared to WT mice (Figure 5L). These findings indicate that fibroblast-specific CD248 deletion impairs PD-L1-mediated immune escape and enhances anti-tumor immunity *in vivo*.

### 3.7 Fibroblast-specific *CD248* gene knockout increases tislelizumab therapeutic efficiency *in vivo*

**FIGURE 6:**
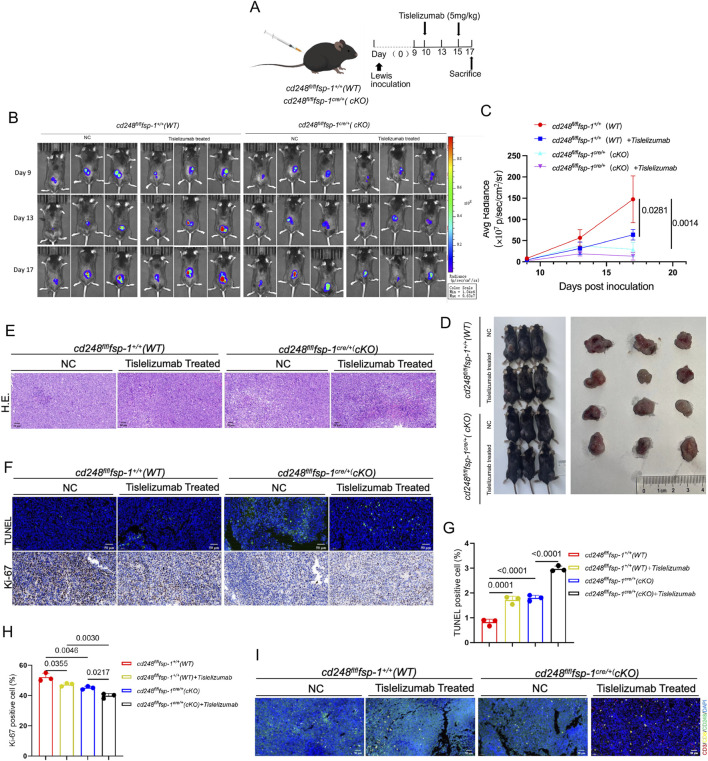
Fibroblast-specific *CD248* gene knockout increased tislelizumab therapeutic efficiency *in vivo*. Overall, 1 × 10^6^ LLC cells/200 μL were administered into *cd248* ^*fl/fl*^ *fsp- 1* ^*+/+*^ (WT) and *cd248* ^*fl/fl*^ *fsp- 1* ^*cre/+*^ (cKO) mice to develop a subcutaneous tumor-bearing model (n = 3 per cohort), then, the mice were given tislelizumab (5 mg/kg). **(A)** A schematic diagram of the model. **(B)** Tumor fluorescence in each group was monitored via BLI. **(C)** The average bioluminescence intensity of each group of mice was assessed. Values are mean ± SEM. **(D)** Mice in each group were euthanized, and tumors were imaged. **(E)** Tumor tissues, as assessed using H&E staining. Scale bar, 50 μm. **(F)** IF for TUNEL (green) staining, and IHC for Ki-67 staining in each cohort. Scale bar, 50 μm. **(G,H)** TUNEL-positive, and Ki-67-positive cells were quantified. Values are mean ± SEM. **(I)** IF staining results show CD3 (red), CD8 (yellow), CD248 (green), and PD-L1 (cyan) in mouse tumors: scale bar, 50 μm.

To further investigate whether CD248 knockout in CAFs enhances the efficacy of ICIs, LLC cells were subcutaneously implanted into *cd248* ^*fl/fl*^ *fsp-1* ^*+/+*^ (WT) and *cd248* ^*fl/fl*^ *fsp-1* ^*cre/+*^ (cKO) mice, followed by administration of the anti-PD-1 antibody tislelizumab to each group (Figure 6A). Tumor progression was monitored using BLI, which showed significantly lower fluorescence intensity in the tislelizumab-treated cKO mice compared to both untreated and tislelizumab-treated WT mice (*p = 0.0281*) (Figures 6B–D). Histological analysis of paraffin-embedded tumor sections using H&E staining revealed necrotic lesions in the tislelizumab-treated tumors (Figure 6E). TUNEL staining demonstrated increased apoptosis in tumors from tislelizumab-treated cKO mice. The Ki-67 staining indicated reduced tumor cell proliferation in this group compared to others (*p = 0.0217*) (Figures 6F–H).

Immunofluorescence staining for CD248, PD-L1, CD3, and CD8 in tumor sections showed reduced CD248 and PD-L1 expression in both untreated and treated cKO mice, along with greater CD8^+^ T cell infiltration in the tislelizumab-treated cKO group (Figure 6I). These results suggest that fibroblast-specific CD248 deletion significantly enhances the therapeutic efficacy of tislelizumab by promoting anti-tumor immune responses *in vivo*.

### 3.8 Augmented CD248 and PD-L1 expressions in NSCLC patients are strongly linked to tumor progression

**FIGURE 7:**
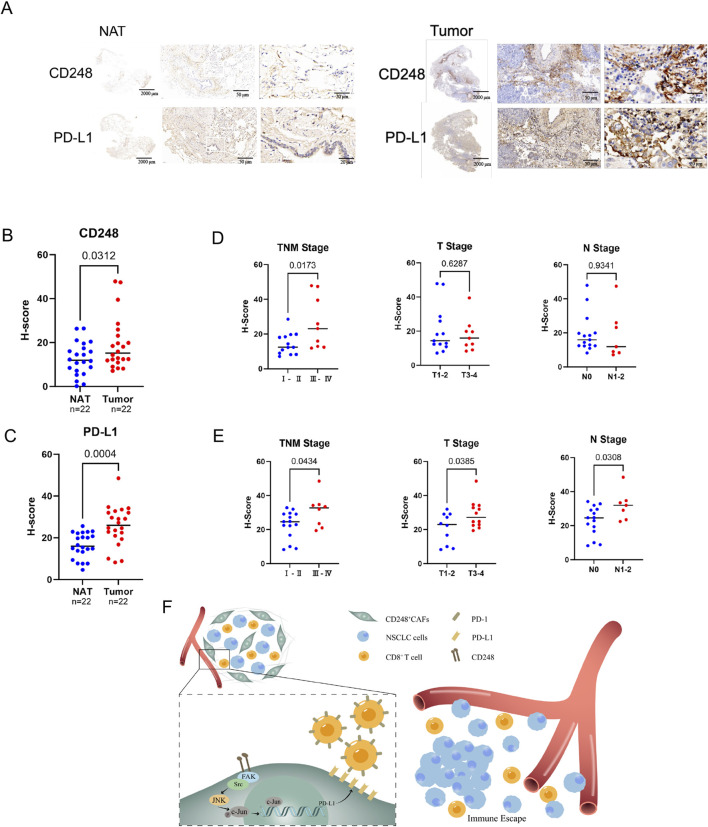
Analysis of CD248 and PD-L1 contents in clinical samples from NSCLC patients. **(A)** Typical immunohistochemical (IHC) CD248 and PD-L1 images from NSCLC biopsies and NAT (n = 22). Scale bars: right: 20 μm, middle: 50 μm, left: 2000 μm. **(B)** The H-scores of CD248 in NSCLC tissues and NAT were calculated. Values are mean ± SD. **(C)** H-scores of PD-L1 in NSCLC tissues and NAT were calculated. Values are mean ± SD. **(D)** The associations between CD248 content and NSCLC clinicopathological features were determined. Values are mean ± SD. **(E)** Potential interactions between PD-L1 content and NSCLC clinicopathological features were analyzed. Values are mean ± SD. **(F)** An illustration of the mechanism: CD248 activates FAK/Src/JNK/c-Jun, inducing PD-L1 expression on CAFs, which promotes NSCLC immune escape.

Tumor tissues and matched NAT were obtained from NSCLC patients and subjected to immunohistochemical staining for CD248 and PD-L1. The analysis demonstrated significantly elevated expression of CD248 (*p = 0.0312*) and PD-L1 (*p = 0.0004*) in NSCLC tissues compared to NAT (Figures 7A–C). Furthermore, the pathological assessment revealed that higher levels of CD248 (*p = 0.0173*) and PD-L1 (*p = 0.0434*) were positively correlated with the advanced tumor–node–metastasis (TNM) stage (Figures 7D,E). Collectively, these findings support a model in which CD248 activates the FAK/Src/JNK/c-Jun signaling cascade, leading to PD-L1 upregulation in CAFs and facilitating immune escape in NSCLC (Figure 7F).

## 4 Discussion

LC is the greatest contributor to cancer-related fatalities globally (Thai et al., 2021). CAFs expressing PD-L1 promote the development of LC (Kawasaki et al., 2023; Ti et al., 2021). In recent years, emerging evidence revealed that CAFs can enhance tumor cell survival and growth and help tumors evade immune surveillance by suppressing immune cell functions, particularly CD8^+^ T cells (Franzese et al., 2024). CD8^+^ T cells contribute significantly to anti-tumor activity by efficiently identifying and killing tumor cells expressing specific antigens. However, the functions of these cells are often suppressed in the TME. TGF-β can block their growth and function (Zhao et al., 2021), while PGE2 can induce apoptosis in the cells (Sinha et al., 2007). Further, CAFs can restrict the survival and growth of CD8^+^ T cells by depleting key nutrients, namely, amino acids and glucose (Zheng et al., 2023). CAFs can also alter the TME structure by secreting many extracellular matrix components, such as collagen and fibronectin (Laurito et al., 2021). However, CAFs exhibit clear heterogeneity (Chen and Song, 2019), and biomarkers of CAFs from various sources differ. A previous study has reported that CD248 was highly expressed in CAFs and promoted NSCLC progression. CD248^+^CAFs express PD-L1 and can block immune escape mediated by CD8^+^ T cells.

PD-L1 interacts with PD-1 to induce cancer immune escape (Yi et al., 2021), and PD-L1 strongly contributes to tumor immune evasion (Cui et al., 2023). ICIs is a cancer immunotherapy strategy that enables immune-mediated tumor targeting by inhibiting immune checkpoint molecules on tumor cell surfaces (Li et al., 2023). However, the limited efficacy of ICIs has become an emerging challenge in recent years. Identifying patient subsets most likely to benefit, such as those with oligometastatic disease, remains a critical area of investigation (Torresan et al., 2025). Our study observed that tislelizumab treatment was more effective in CD248 knockout mice. In treatment assay, the apparent discordance between the significant reduction in fluorescent signal intensity and the less pronounced difference in gross tumor morphology likely reflects the more rapid decline in tumor cell viability activity induced by Tislelizumab treatment, preceding substantial resolution of the physical tumor mass. Consistent with the potential of combinatorial approaches, studies on immunotherapy-based combinations in metastatic NSCLC have demonstrated enhanced anti-tumor responses by simultaneously targeting stromal and immune pathways (Desai and Peters, 2023). These findings suggest that eliminating CD248 expression in CAFs can enhance the therapeutic response to ICIs. While CD248 orchestrates multiple pro-tumorigenic programs, its role as a signaling hub necessitates time-resolved studies in dynamic TMEs. Future work will employ spatial transcriptomics to map pathway dominance across NSCLC stages and test combinatorial targeting strategies in the context of emerging therapeutic targets for NSCLC (Liu et al., 2024).

Recent studies have highlighted that signaling pathways such as BRD4/IRF1 (Hogg et al., 2017), PI3K/AKT, and FAK/Src contribute to PD-L1 upregulation (Wang et al., 2022b), facilitating tumor immune escape. FAK/Src activation can trigger downstream signaling involving JNK and c-Jun. Our study demonstrated that CD248 enhances the p-FAK, p-Src, p-JNK, and p-c-Jun and promotes the nuclear translocation of c-Jun, leading to increased PD-L1 expression on CAFs. On the other hand, treatment with the inhibitors PF-562271, dasatinib, and SP600125 significantly reduced the expression of p-FAK, p-Src, p-JNK, p-c-Jun, and PD-L1. These findings suggest that CD248 activates the FAK/Src/JNK/c-Jun pathway to induce PD-L1 expression on CAFs, contributing to immune escape in NSCLC. However, several limitations remain unaddressed. The precise molecular mechanisms by which CD248 drives PD-L1 expression through this axis and how this modulates immune evasion in NSCLC require further investigation. Furthermore, the specific ligands interacting with CD248 have yet to be identified. While our data support CD248-driven FAK/Src/JNK/c-Jun activation as a key regulator of PD-L1 in CAFs, future studies validating c-Jun binding to the PD-L1 promoter and performing pathway rescue experiments will further solidify causality.

In conclusion, this study underscores the pivotal role of CD248 expression on CAFs in driving PD-L1 upregulation, therefore facilitating immune escape in NSCLC. Our findings also shed light on key signaling pathways involving CD248^+^ CAFs that contribute to tumor immune escape and propose a potential therapeutic strategy targeting this axis for improved NSCLC treatment outcomes.

## Data Availability

The original contributions presented in the study are included in the article/Supplementary Material, further inquiries can be directed to the corresponding authors.

## Abbreviations

CAFs, cancer-associated fibroblasts; NAT, normal adjacent tissues; NC, negative control; NFs, normal fibroblasts; NSCLC, non-small cell lung cancer; STR, short tandem repeat; TME, tumor microenvironment.
