# Supplementary material for: CD248 induces PD-L1 expression on cancer-associated fibroblasts to promote NSCLC immune escape
Source: Front Cell Dev Biol. 2025 Jul 15;13:1635915. doi: 10.3389/fcell.2025.1635915 (PMC12304000; doi:10.3389/fcell.2025.1635915)
Supplement: Supplementary file 1 [file Table1.docx]

**Supplementary Table 1. Antibodies and manufacturers**

| Antibody | Company | Catalog No. | Concentrations |
| --- | --- | --- | --- |
| CD248 | CST | #47948 | Western blot: 1:1000, IF:1:400 |
| FAP | Servicebio | #GB11096 | Western blot: 1:1000 |
| α-SMA | Servicebio | #GB11044 | Western blot: 1:1000 |
| α-SMA | Servicebio | #GB111364 | IF:1:500 |
| FAK | Absin | #131894 | Western blot: 1:500 |
| Src | Servicebio | #GB11783 | Western blot: 1:1000 |
| phospho-Src | Absin | # abs130790 | Western blot: 1:1000 |
| JNK | CST | #9252 | Western blot: 1:1000, IF: 1:200 |
| c-Jun | CST | #9165 | Western blot: 1:1000 |
| PD-L1 | CST | #86744 | Western blot: 1:1000, IF: 1:200 |
| phospho-FAK | CST | #8556 | Western blot: 1:1000 |
| phospho-JNK | CST | #4668 | Western blot: 1:1000 |
| phospho-c-Jun | CST | #91952 | Western blot: 1:1000 |
| GAPDH | Servicebio | #11002 | Western blot: 1:2000 |
| FAK | Santa Cruz | sc-271195 | IP:1:500 |
| TEM-1(CD248) | Santa Cruz | sc-377221 | IF:400 |
| HRP-goat anti-abbit | Servicebio | #GB23303 | Western blot:1:5000 |
