# Supplementary material for: CD248 induces PD-L1 expression on cancer-associated fibroblasts to promote NSCLC immune escape
Source: Front Cell Dev Biol. 2025 Jul 15;13:1635915. doi: 10.3389/fcell.2025.1635915 (PMC12304000; doi:10.3389/fcell.2025.1635915)
Supplement: Supplementary file 2 [file Table2.docx]

**Supplementary Table 2**

**Primer sequence for q-PCR**

| gene name | Forward primer | Reverse primer |
| --- | --- | --- |
| Human *CD248* | 5’-ACTACGTTGGTGGCTTCGAG-3’ | 5’-CACTGAGGAGTGGTAGGGGA-3’ |
| Human *PD-L1* | 5’-TGGCATTTGCTGAACGCATTT-3’ | 5’-TGCAGCCAGGTCTAATTGTTTT-3’ |
| Human *GAPDH* | 5’-GGAGCGAGATCCCTCCAAAAT-3’ | 5’-GGCTGTTGTCATACTTCTCATGG-3’ |
