# Supplementary figures and images for: CD248 induces PD-L1 expression on cancer-associated fibroblasts to promote NSCLC immune escape
Source: Front Cell Dev Biol. 2025 Jul 15;13:1635915. doi: 10.3389/fcell.2025.1635915 (PMC12304000; doi:10.3389/fcell.2025.1635915)

Fig 1 G

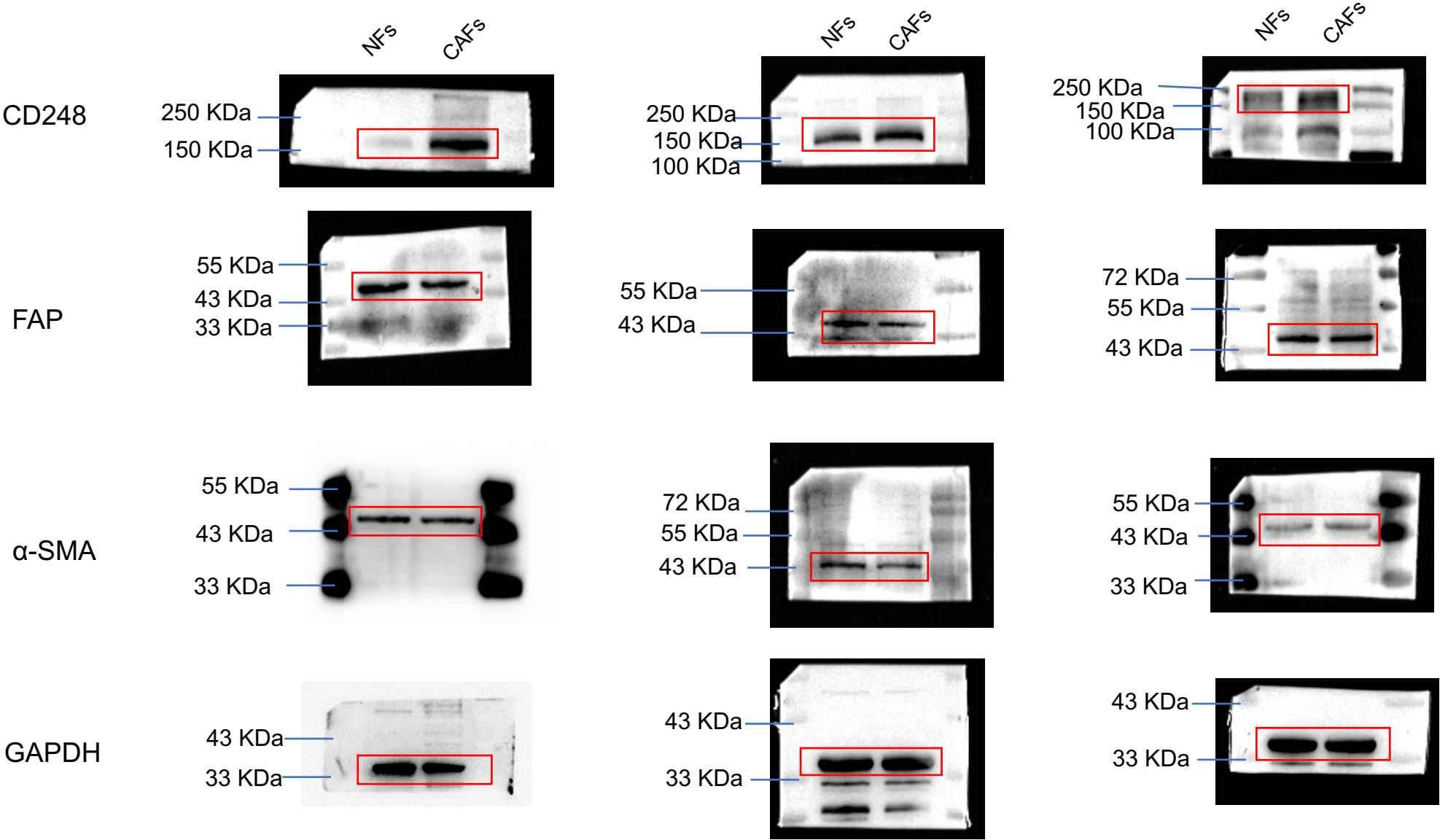

Fig 2 C

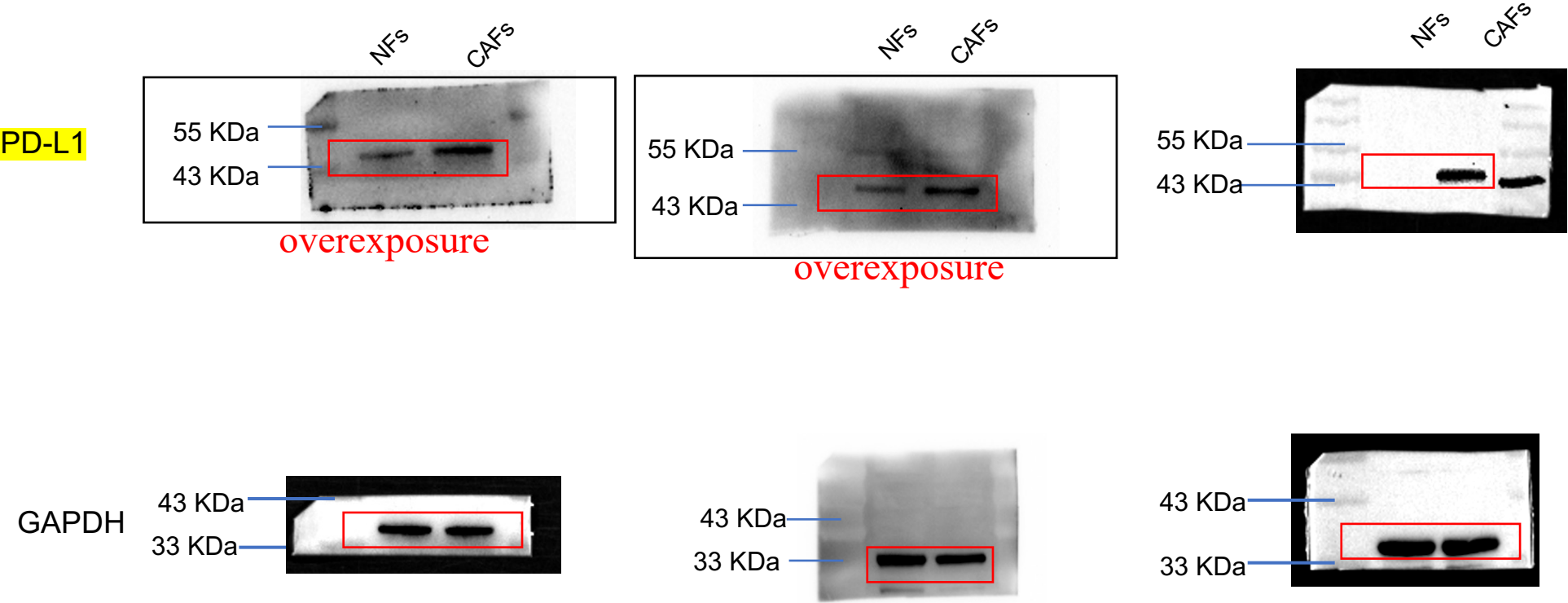

Fig 2 C

PD-L1

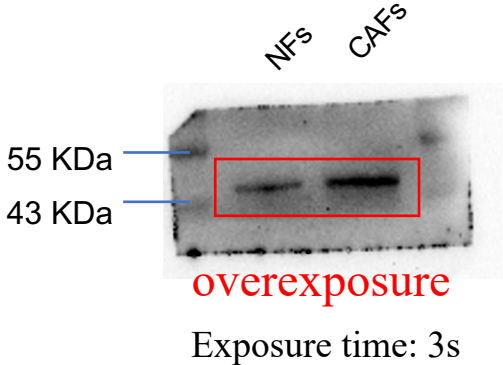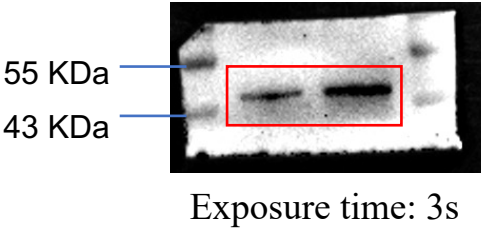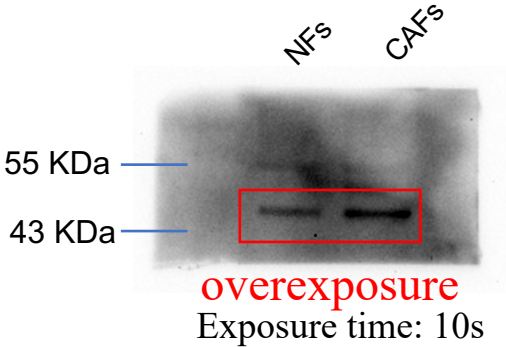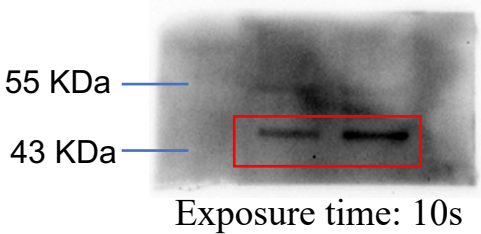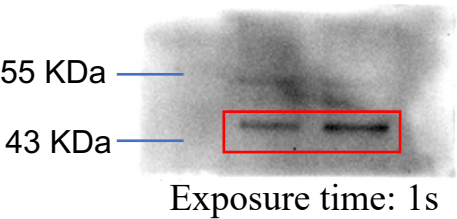

Fig 2 E

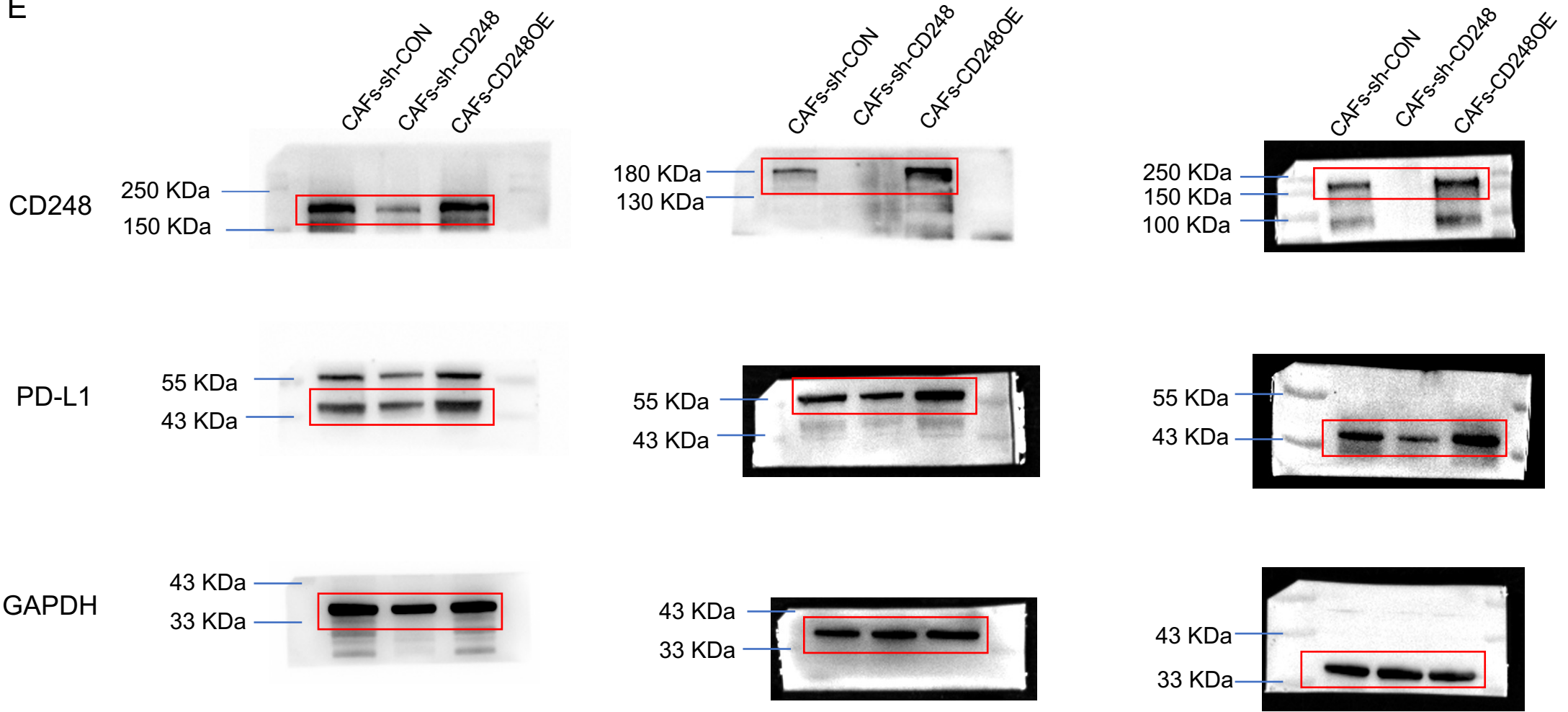

Fig 4A

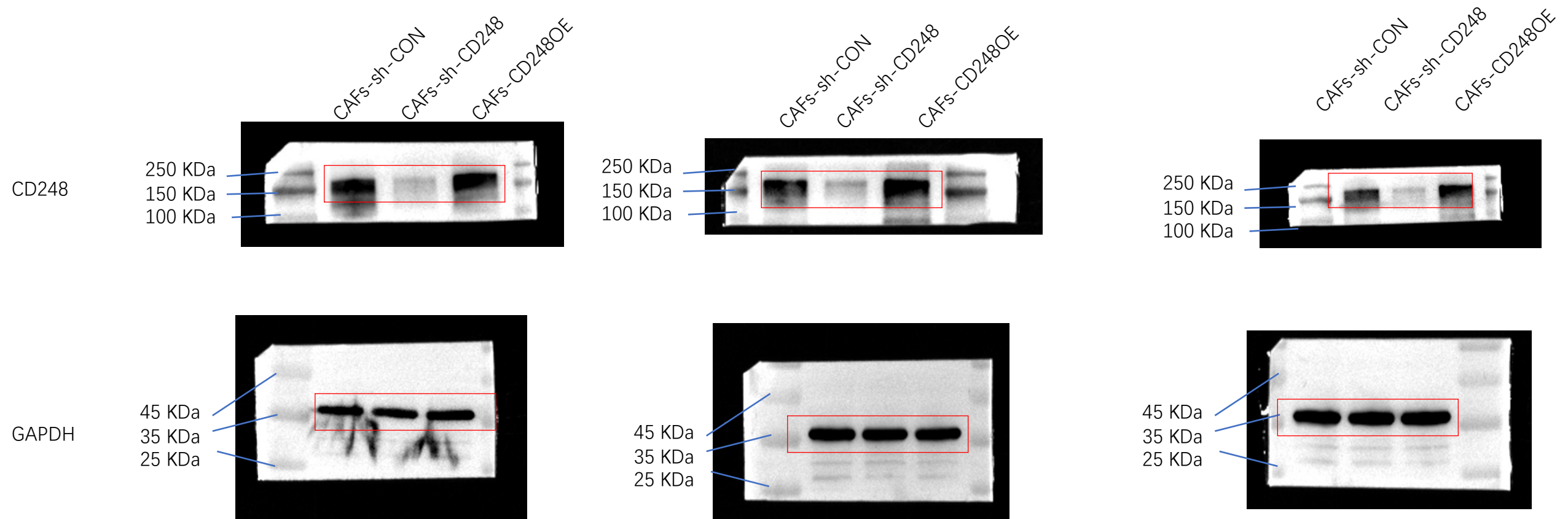

Fig 4 A

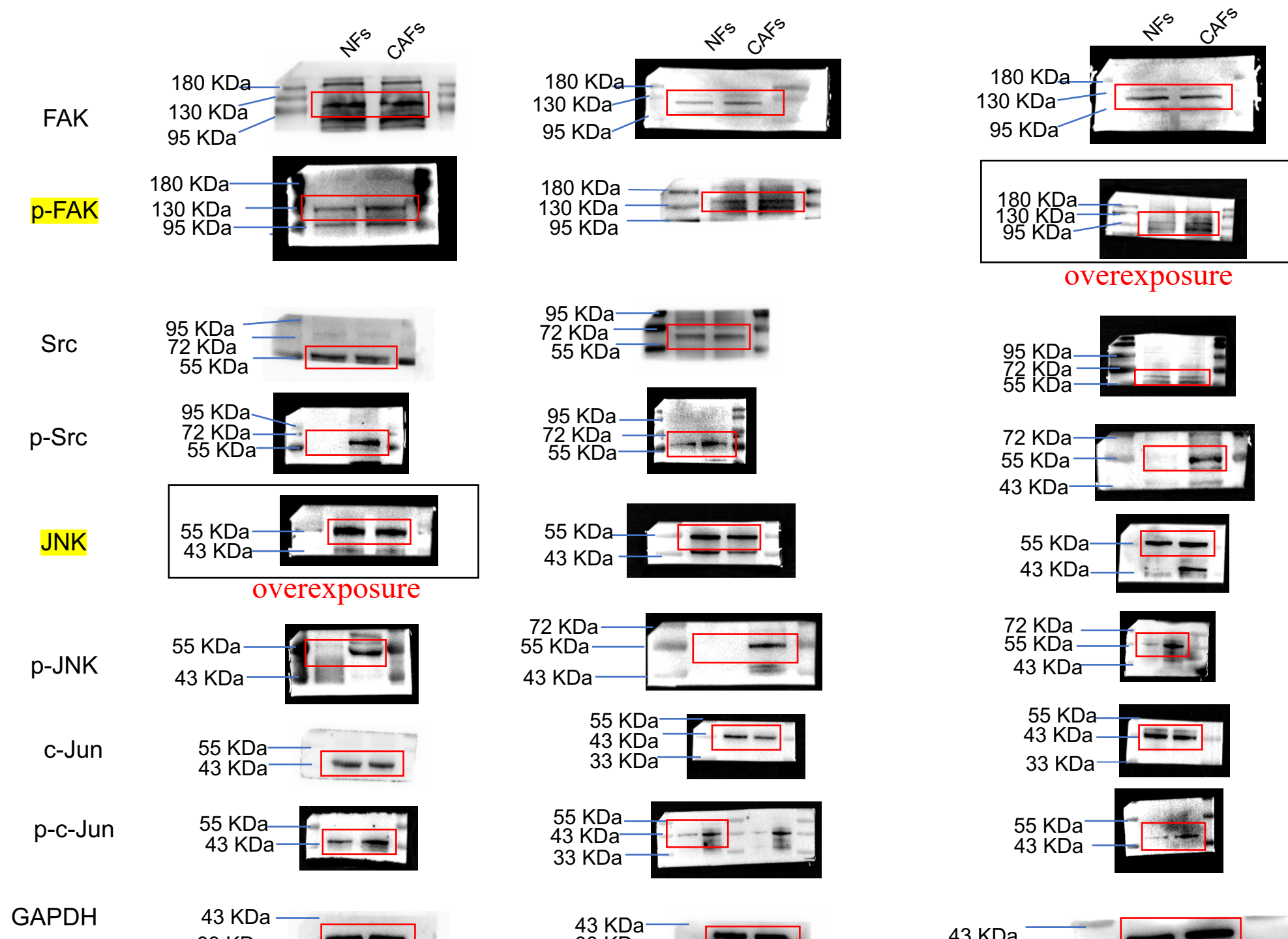

Fig 4 A

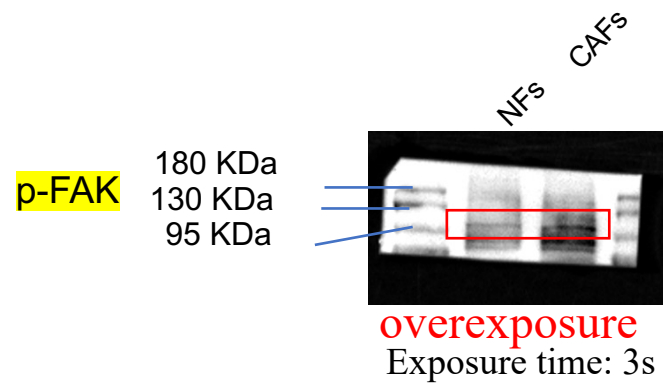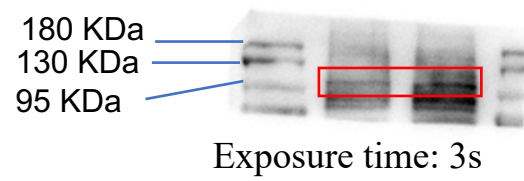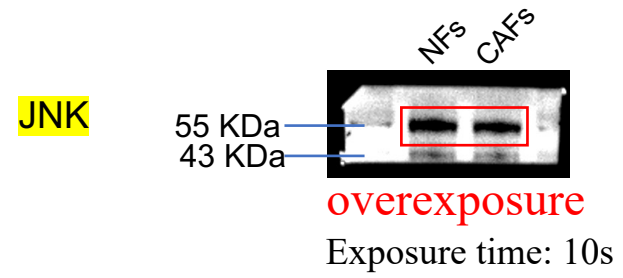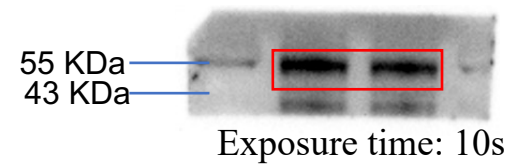

Fig 4B

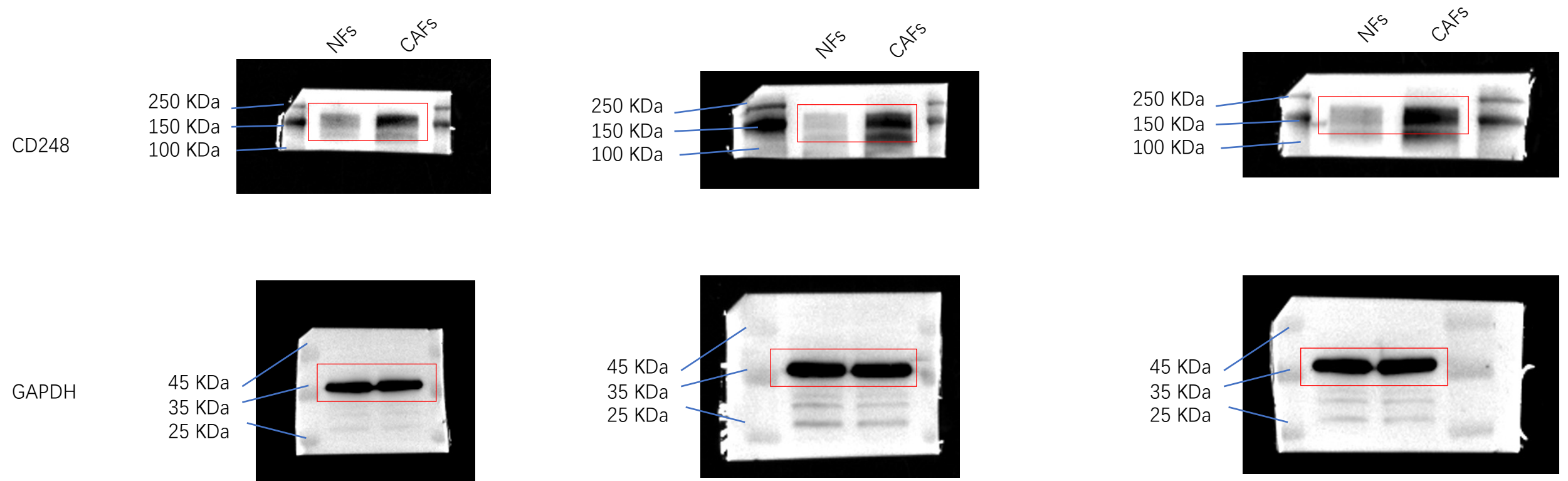

Fig 4 B

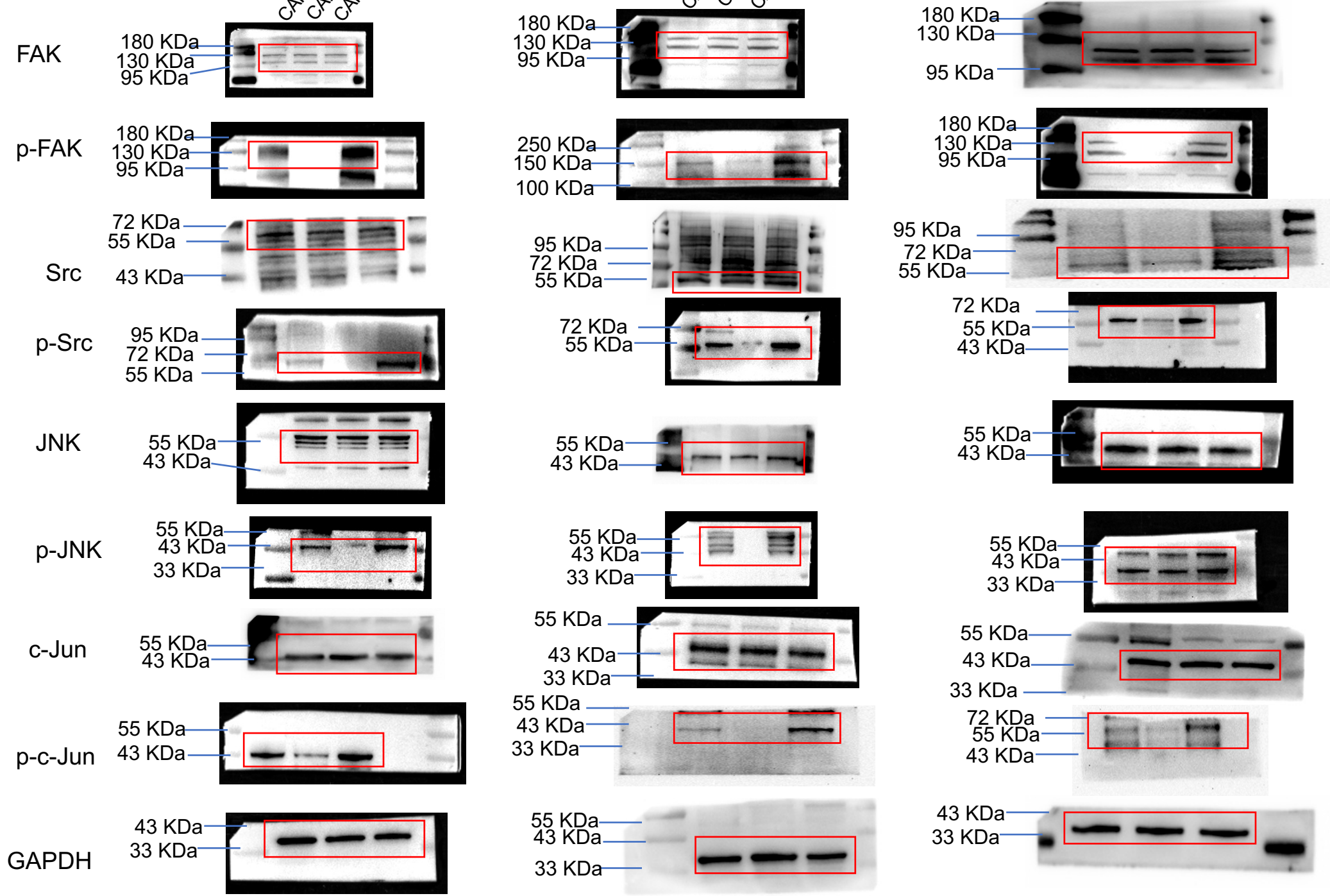

Fig 4D

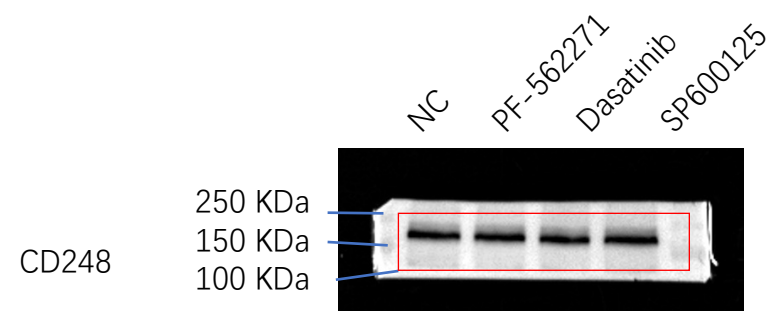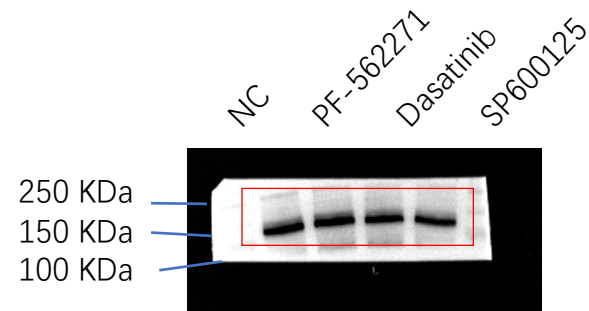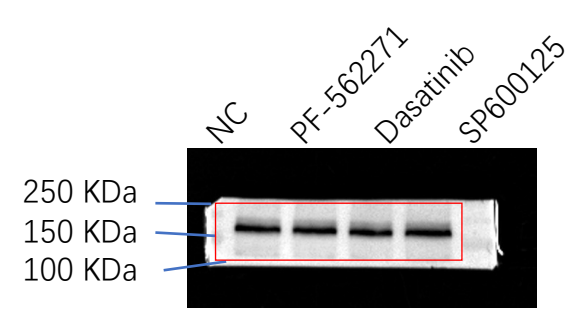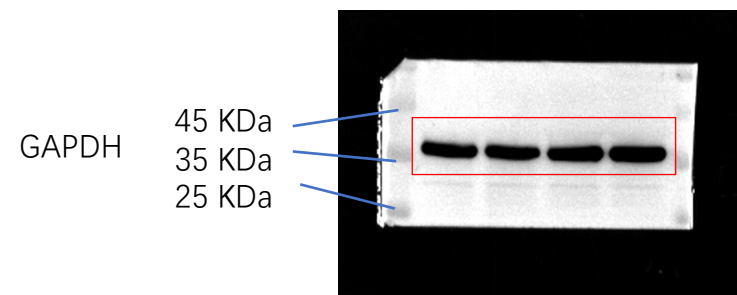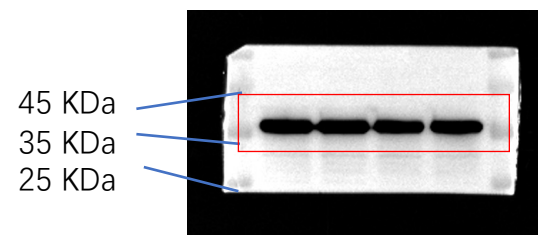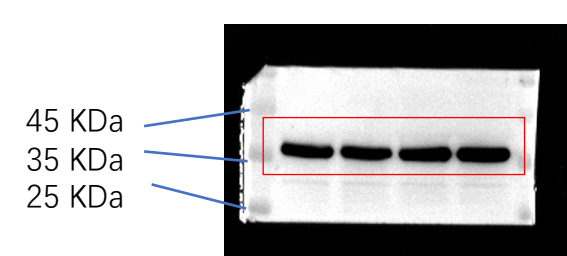

Fig 4 D

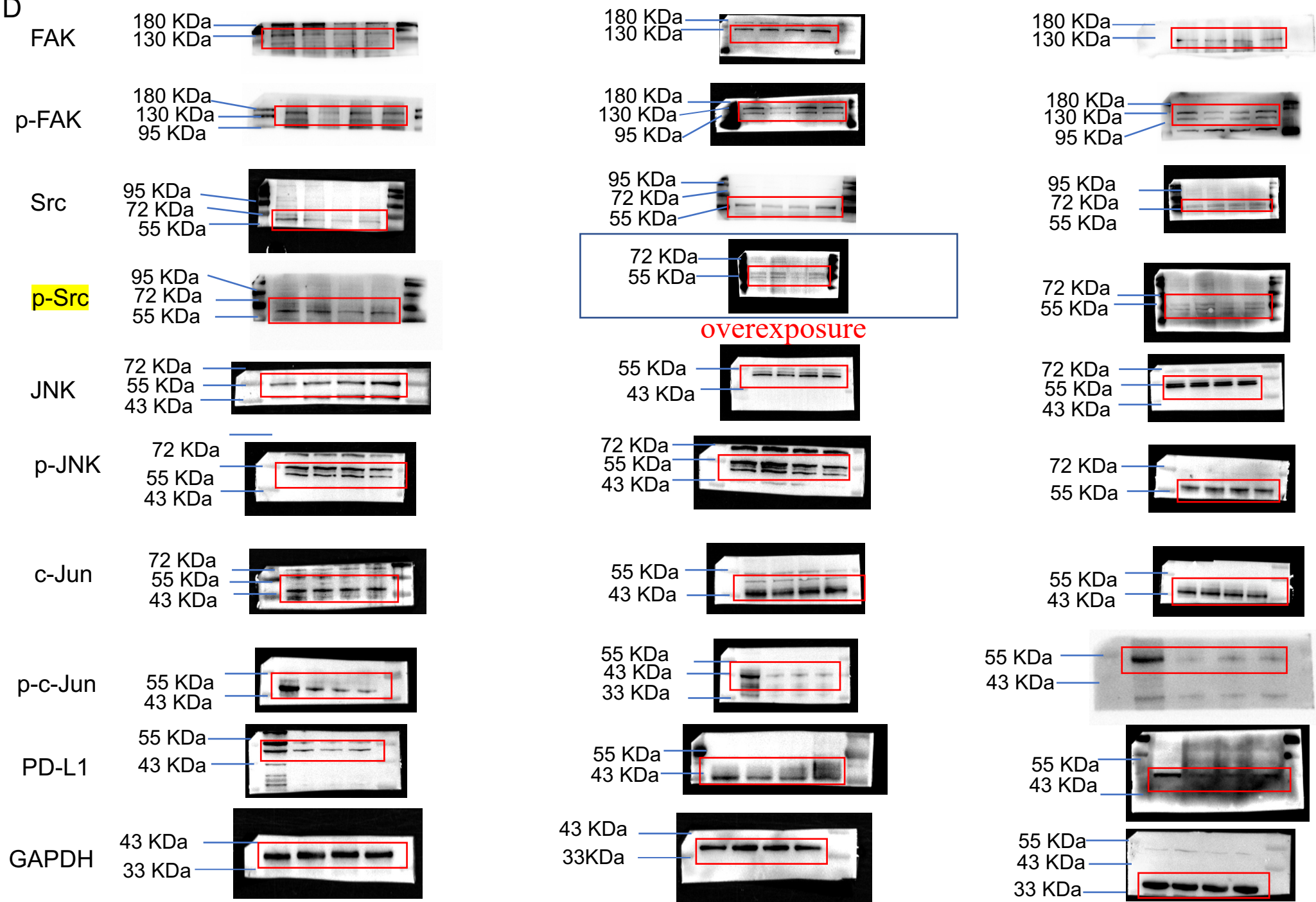

Fig 4 D

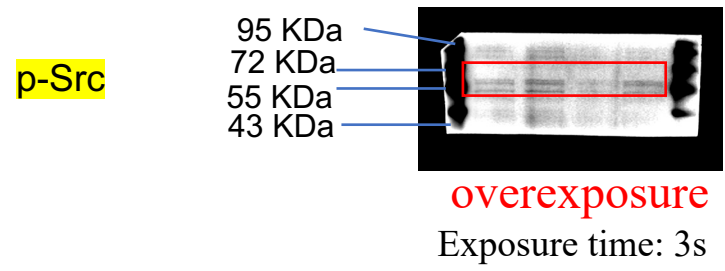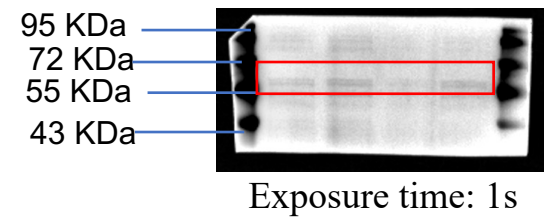

Fig 4 G

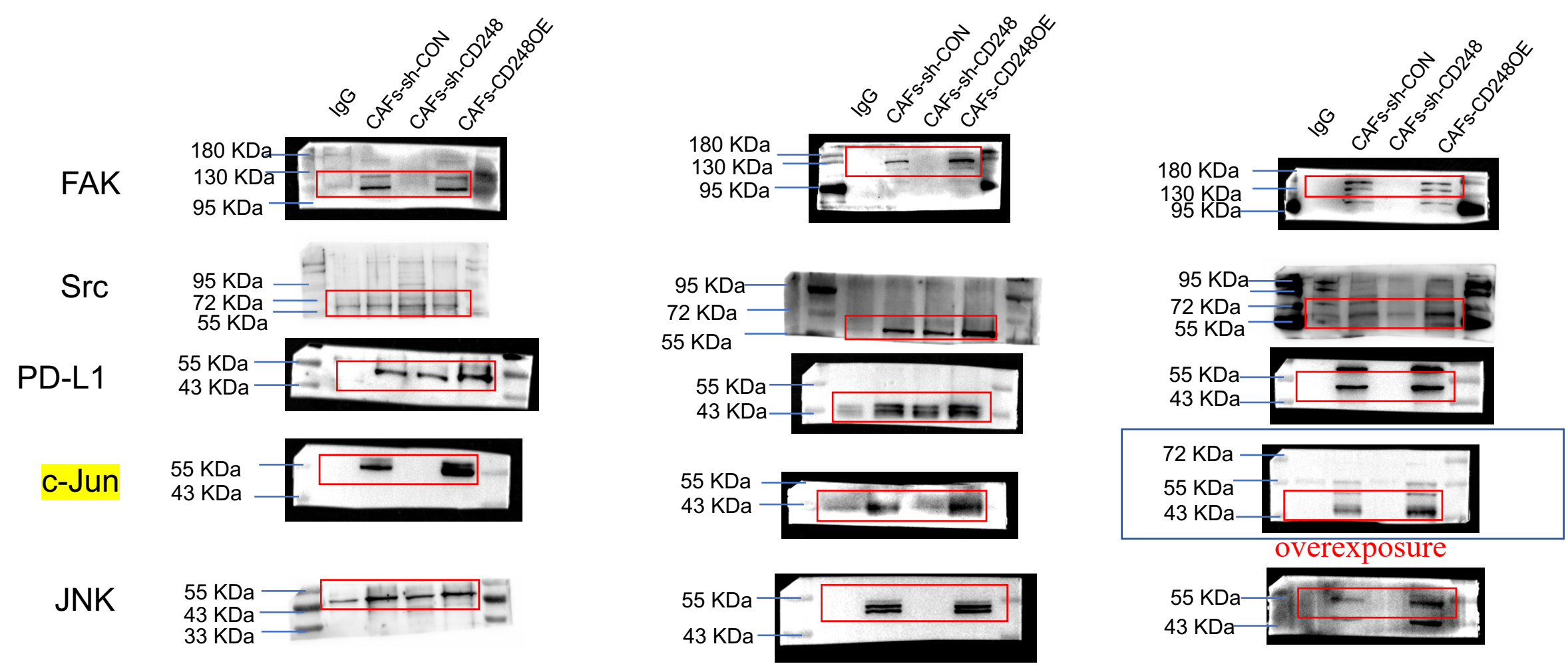

Fig 4 G

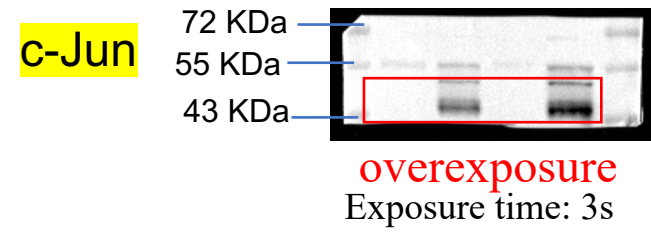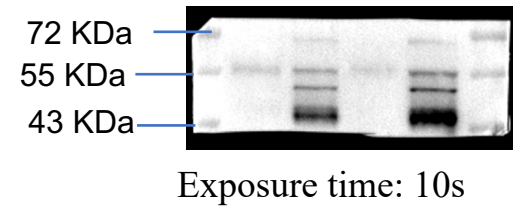

Fig 4 G

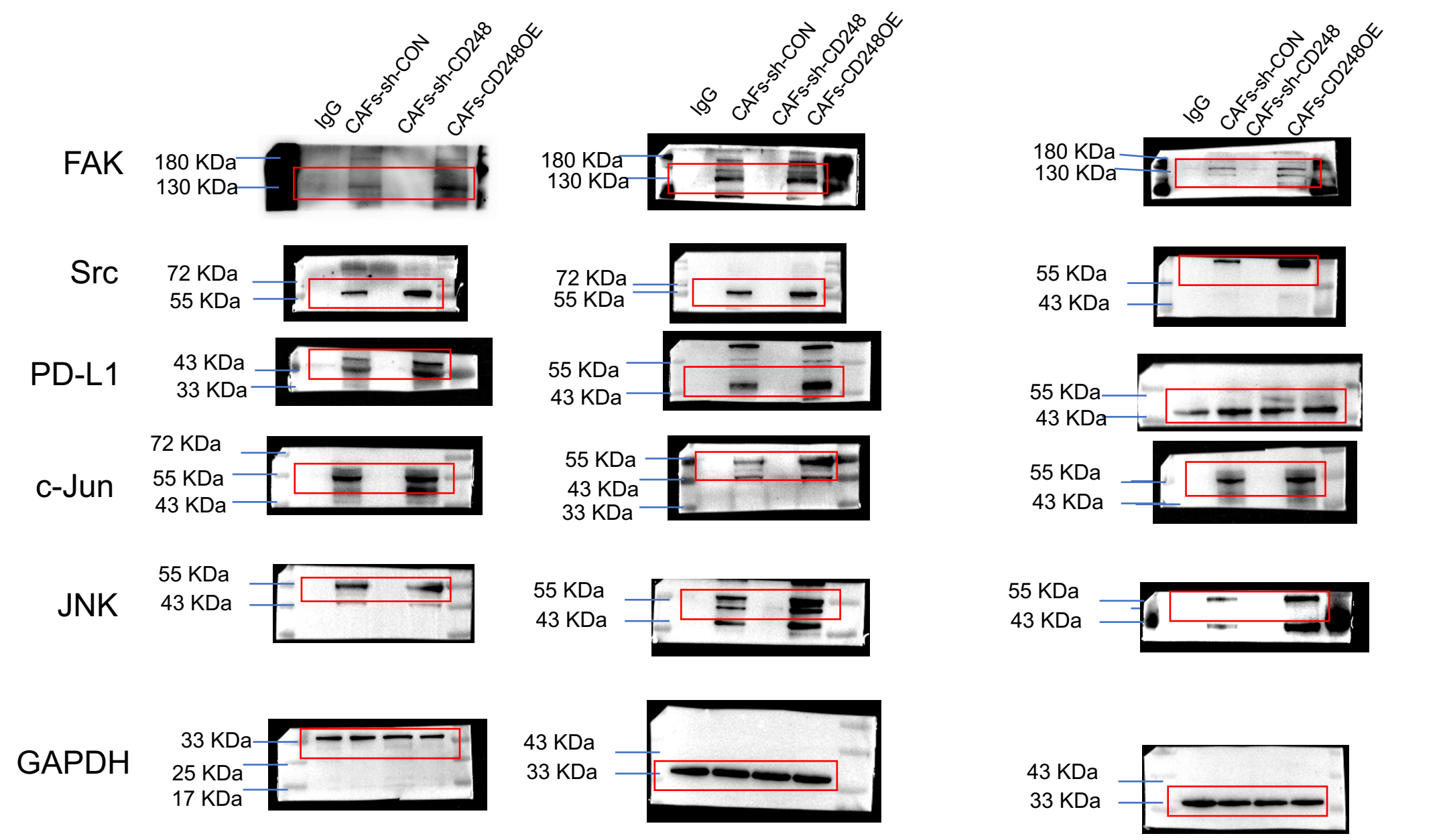

Fig 5L

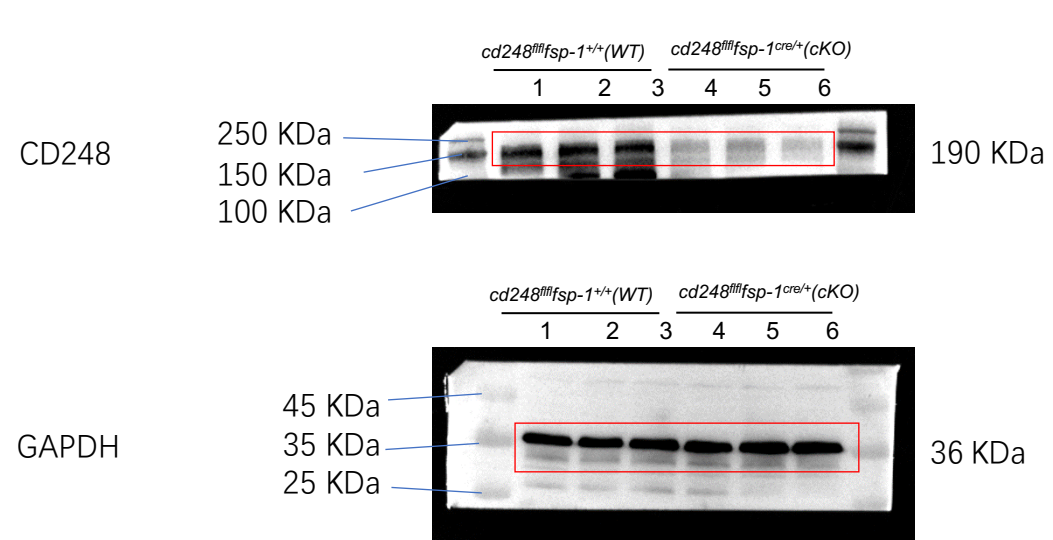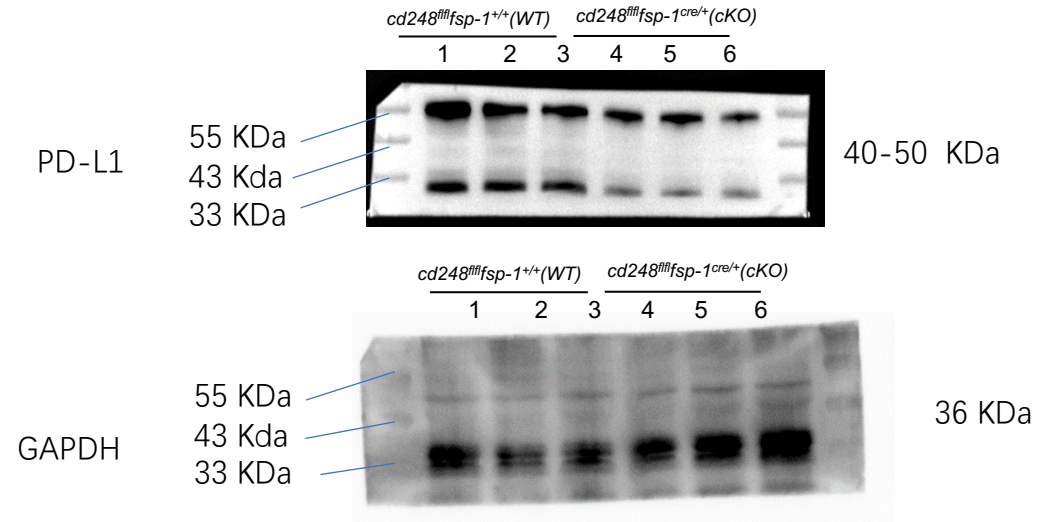

Supplement: Supplementary file 3 [file DataSheet1.pdf]
